# Public Awareness of the Symptoms and Risk Factors of Thyroid Disease in Saudi Arabia

**DOI:** 10.7759/cureus.71256

**Published:** 2024-10-11

**Authors:** Ramy H Agwa, Warda Othman, Khalid M Alkhalifah, Reem M Alharthi, Fatemah H Algafli, Sara M Alghamdi, Taif S Alghamdi, Sarah I Alghamdi, Salman J Alharthi

**Affiliations:** 1 Division of Hepatology and Gastroenterology, Department of Internal Medicine, Mansoura University, Mansoura, EGY; 2 Division of Hepatology and Gastroenterology, Department of Internal Medicine, Al-Baha University, Al-Baha, SAU; 3 Division of Hepatology, Department of Internal Medicine, Menoufia University, Shebin El Kom, EGY; 4 Department of Ear, Nose, and Throat, Al-Rass General Hospital, Qassim Health Cluster, Al-Rass, SAU; 5 Faculty of Medicine, Al-Baha University, Al-Baha, SAU; 6 Department of Internal Medicine, King Fahad Hospital, Al-Baha, SAU

**Keywords:** al-baha, ksa, risk factors, thyroid disease, thyroid hormone

## Abstract

Introduction: Thyroid disorders are a common manifestation of hormonal disorders that result from either excessive or insufficient production of thyroid hormones and the swelling of the thyroid gland. A lack of information and awareness of the signs and risk factors of thyroid problems can lead to untreated patients, thereby harming public health.

Methods: Observational cross-sectional research was conducted in the Al-Baha region of Saudi Arabia, with 531 residents selected from the general population. The data were analyzed using SPSS to derive significant insights, and questionnaires were used to collect the data.

Results: The results revealed that 79 (14.9%) of the participants had been diagnosed with thyroid disease. The risk factors for thyroid diseases reported were as follows: female gender (386, 72.7%); exposure to radiation (329, 62.0%); smoking (312, 58.8%); family history (300, 56.5%); and insufficient or excess intake of iodine (277, 52.2%). Only 110 (20.7%) of the participants knew that the use of amiodarone medication is a risk factor for thyroid diseases. The symptoms reported included fatigue (430, 81.0%), feeling cold, and weight gain (409, 77.0%); neck lump (379, 71.4%); depression (367, 69.1%); and hair loss (336, 63.3%). Early diagnosis (426, 80.2%), a well-exercised program (394, 74.2%), and a well-balanced diet (392, 73.8%) were the prevention methods reported by a majority of the participants. The study results revealed an average score of 13.57±2.11, with a minimum knowledge score of 0 and a maximum of 25. A statistically significant difference was observed in knowledge scores based on the participant's gender, age, education level, previous diagnosis with thyroid diseases, family history of thyroid diseases, and having performed thyroid gland investigation with p-values <0.05 (<0.001, 0.019, 0.027, <0.001, 0.023, and 0.031), respectively.

Conclusion: Nearly half of the participants had low knowledge scores regarding the risk factors and clinical manifestations of thyroid diseases. Although a sizable proportion of participants demonstrated awareness of some clinical presentations and prevention methods, there are serious knowledge gaps regarding the risk factors and symptoms of thyroid disease among the general population in the Al-Baha region. There is a need for targeted health education programs to improve public awareness of risk factors and early detection and prevention measures of thyroid diseases to improve the quality of life among community members.

## Introduction

Thyroid diseases drastically influence the metabolism, growth, and development of the body. They are a global health problem affecting the lives of people worldwide [1]. The thyroid gland controls numerous physiological functions, specifically the hormones it secretes. A slight disturbance in the gland's functions can result in significant health challenges, such as hyperthyroidism or hypothyroidism, which affects approximately 1.3% of individuals worldwide [2,3]. Hypothyroidism occurs in two forms: primary, which, in this context, is from the gland itself, and secondary, which occurs together with other conditions. Hypothyroidism is a condition characterized by the inadequate secretion of thyroid hormones [3]. This condition is further manifested in the Al-Baha region of Saudi Arabia, where the prevalence of thyroid diseases is stunningly high at 16%, primarily in women [4]. Yet, despite the incredible importance of the thyroid in well-being, an awareness gap exists. Thyroid illnesses are the most underdiagnosed health problem globally [5]. Such an awareness gap is particularly worrying in Saudi Arabia: General knowledge is average at best in such conditions, while understanding the specific risk factors is still rarer [4]. This knowledge gap creates a significant disadvantage by delaying the diagnosis and treatment of thyroid disorders, thereby impeding timely intervention that could prevent serious health complications [6]. For example, several studies in other regions of Saudi Arabia, such as in the Eastern and Central provinces, have consistently noted that although the prevalence of a lack of awareness about thyroid disorders is relatively high, a lot of people are increasingly becoming victims of the same [7]. It was established that, although studies on the evaluation of awareness and knowledge by the residents of Saudi Arabia are scanty, they are relatively low at 31% among the residents of Riyadh, who had only heard of the disease [8]. It was even lower in Jeddah at 28%, who had a considerably good knowledge of thyroid disease [9]. Hypothyroidism is associated with neuropsychiatric effects; severe hypothyroidism is related to cognitive malfunctions such as mood disorders, dementia, and depression [10]. This association of neuropsychiatric clinical manifestations with thyroid disorders presents a significant risk to a patient's quality of life. So far, no detailed studies have been conducted to gauge the level of awareness among the residents precisely and the diversity of factors that influence the knowledge of the signs and risk factors of thyroid diseases in the Al-Baha region. This study will try to fill in the knowledge gap and create awareness.

## Materials and methods

Study design

This research utilized a cross-sectional design and was carried out between November 2023 and April 2024. It involved the citizens of Al-Baha, Saudi Arabia, and aimed to evaluate their knowledge of thyroid disease manifestations and risk factors.

Sample size and sampling technique

The Epi Info Version 7.2.2.6 (Centers for Disease Control and Prevention, Atlanta, GA, USA) was employed to determine the sample size for this study. The minimum required sample size was 384 participants, considering the population of the Al-Baha region (339,174), 80% power, and a 95% confidence interval (CI). Also, the expected frequency was 50%. To enhance the precision of the results, the sample size was increased to 531 responses. During the data collection period, participants were selected using a non-probability convenience sampling technique based on their availability and willingness to participate. This method facilitated the efficient enlistment of participants; however, it suggested an increased sample size to account for potential biases and enhance the accuracy of the findings.

Inclusion and exclusion criteria

The study included all the above 18 years of age of Al-Baha, irrespective of gender, who expressed willingness to participate. The study excluded the non-residents of Al-Baha, minors or people below the age of 18, and those who did not consent to be part of the study.

Data collection tools and procedures

Using a previously validated questionnaire from Alyahya et al. [5], we adapted the tool to align with the goals and objectives of our research. The reliability and validity of the original questionnaire were confirmed using the Cronbach's alpha test, as reported in the prior study. A pilot test was conducted with 10 respondents, distinct from the main study participants, to assess its feasibility, understandability, and readability. Based on the feedback from the pilot study, minor adjustments were made to the questionnaire. The final version was disseminated online via WhatsApp, Telegram, and Facebook groups to residents of the Al-Baha region. The questionnaire consisted of three sections: the first focused on sociodemographic data, the second on the participants' past history of thyroid disease, and the third on their knowledge regarding risk factors, manifestations, and preventive behaviors. The scoring system included 25 questions, with the score derived from the responses to the second and third sections. Based on the total score, participants were categorized into three groups: low knowledge level (total score below 50%), moderate knowledge level (50%-75%), and high knowledge level (above 75%).

Data analysis

The acquired data were input into Microsoft Excel spreadsheets (Microsoft Corp., Redmond, WA, USA) for data cleaning purposes. This approach entailed identifying and removing duplicate entries, identifying any outliers, and confirming the presence of any missing data points. The data were later encoded and analyzed using IBM SPSS Statistics for Windows, Version 27.0 (Released 2020; IBM Corp., Armonk, NY, USA). Categorical variables were represented by counts and frequencies, while continuous variables were expressed as means and standard deviations. The association between categorical variables was assessed using the chi-square test, which is a form of inferential statistics. The significance level was set at a p-value of less than 0.05. The main objective of this thorough analytical technique was to extract meaningful insights and detect patterns within the dataset.

Ethical considerations

Ethical approval for this study was secured from Al-Baha University's Committee (REC/MED/BU-FM/2024/7), and several ethical considerations were meticulously addressed. Measures were implemented to uphold confidentiality and ensure informed consent. Participants were comprehensively briefed on the study's objectives, procedures, potential risks, and benefits before participation. Their consent was solicited voluntarily without any coercion. The researchers pledged to safeguard participants' privacy by preserving their anonymity and sensitive data.

## Results

Table 1 shows that a total of 531 participants completed the online questionnaires. The majority of the participants were females (393, 74.0%), with a notable proportion (215, 40.5%) aged 18-28 years, and most of them were Saudi nationals (507, 95.5%). A remarkable proportion of the respondents (231, 43.5%) were residents of the Al-Baha region, with more than half being married (320, 60.3%). Furthermore, the majority (372, 70.1%) had a university-level education, with nearly half being employed (263, 49.6%). 

**Table 1:** Sociodemographic information of the participants (N=531). The data are presented in frequencies (n) and proportion (%).

Sociodemographic information	Category	Frequency and proportion, n (%)
Gender	Female	393 (74.0)
	Male	138 (26.0)
Age (years)	18-28	215 (40.5)
	28-38	88 (16.6)
	38-48	162 (30.5)
	48-58	60 (11.3)
	>58	6 (1.1)
Nationality	Saudi	507 (95.5)
	Non-Saudi	24 (4.5)
Residence area	Al-Baha city	231 (43.5)
	Other cities	300 (56.5)
Marital status	Unmarried	211 (39.7)
	Married	320 (60.3)
Education level	Uneducated	3 (0.5)
	Elementary level	9 (1.7)
	Secondary level	85 (16.0)
	Intermediate	8 (1.5)
	Higher education	54 (10.2)
	University level	372 (70.1)
Employment status	Student	152 (28.6)
	Unemployed	116 (21.8)
	Employed	263 (49.6)

Table 2 depicts the participants’ past medical history of thyroid disorders. The findings revealed that 79 (14.9%) of the participants had been diagnosed with thyroid disease, with hypothyroidism being the most prevalent disorder detected in nearly half (40, 50.6%) of the diagnosed participants. A notable proportion of participants (216, 40.7%) reported a family history of thyroid disease. Less than half of the participants (234, 44.1%) had done thyroid investigation before, with a doctor’s suggestion, 91 (38.9%) being the most frequently mentioned reason for the investigation. A remarkable proportion of the participants (206, 38.8%) reported that thyroid function tests had been performed on them.

**Table 2:** Participants’ past medical history of thyroid disease. The data are presented in frequencies (n) and proportion (%).

Questions	Categories	Frequency and proportion, n (%)
Have you been diagnosed with any thyroid diseases?	Yes	79 (14.9)
	No	452 (85.1)
What type of thyroid disease?	Hyperthyroidism	16 (20.3)
	Hypothyroidism	40 (50.6)
	Thyroid nodule	12 (15.2)
	Thyroid cancer	6 (7.6)
	I don’t know	5 (6.3)
Did one of your family members have any thyroid diseases?	Yes	216 (40.7)
	No	315 (59.3)
Have you done any of thyroid investigations before?	Yes	234 (44.1)
	No	297 (55.9)
Why have you done the investigation?	Routine check	77 (32.9)
	Doctor’s suggestion	91 (38.9)
	Neck swelling	31 (13.3)
	I don’t know	16 (6.8)
	None	19 (8.1)
Which of the following investigations performed for you?	Thyroid function test	206 (38.8)
	Ultrasound of the thyroid gland	91 (17.1)
	I don’t know	234 (44.1)

Table 3 depicts the participants’ knowledge of the risk factors, their manifestation, and the prevention of thyroid diseases. The participants demonstrated adequate knowledge regarding the risk factors for thyroid diseases, including female gender (386, 72.7%), exposure to radiation (329, 62.0%), smoking (312, 58.8%), family history (300, 56.5%), and insufficient or excess intake of iodine (277, 52.2%). Interestingly, less than one-quarter of the participants (110, 20.7%) knew that the use of amiodarone medication was a risk factor for thyroid diseases. Regarding the symptoms, the majority of the participants knew the following clinical manifestations: fatigue (430, 81.0%), feeling cold and weight gain (409, 77.0%), neck lump (379, 71.4%), depression (367, 69.1%), and hair loss (336, 63.3%). Regarding prevention of the disease, the majority of the participants demonstrated good knowledge about early diagnosis (426, 80.2%), a well-exercised program (394, 74.2%), and a well-balanced diet (392, 73.8%) as ways of preventing thyroid diseases and their complications. However, less than half (183, 34.4%) knew that avoiding soy foods prevented thyroid disease among women.

**Table 3:** Participants’ knowledge about the risk factors, manifestation, and preventive behaviors of thyroid diseases. The data are presented in frequencies (n) and proportion (%).

Questions	Categories	Frequency and proportion, n (%)
Knowledge of risk factors		
Do you agree that smoking is associated with a higher chance of developing thyroid diseases?	Yes	312 (58.8)
	No	38 (7.2)
	I don’t know	181 (34.0)
Do you think that being exposed to radiation increases the likelihood of developing thyroid diseases?	Yes	329 (62.0)
	No	30 (5.6)
	I don’t know	172 (32.4)
Do you agree that women are more prone to acquiring thyroid disorders?	Yes	386 (72.7)
	No	26 (4.9)
	I don’t know	119 (22.4)
Do you believe that thyroid diseases are increased risk by an insufficient or excessive intake of iodine?	Yes	277 (52.2)
	No	38 (7.2)
	I don’t know	216 (40.6)
Do you believe that thyroid diseases are increased risk by the pregnancy and postpartum period?	Yes	215 (40.5)
	No	108 (20.3)
	I don’t know	208 (39.2)
Is it your opinion that the medication amiodarone (marketed under the names Pacerone, Cordarone, Advadarone, and Sedacoron) is an increased risk for thyroid diseases?	Yes	110 (20.7)
	No	37 (7.0)
	I don’t know	384 (72.3)
Do you believe that the consumption of lithium is an increased risk for thyroid disorders?	Yes	138 (26.0)
	No	38 (7.1)
	I don’t know	355 (66.9)
Do you believe that thyroid diseases may be inherited from one's family?	Yes	300 (56.5)
	No	87 (16.4)
	I don’t know	144 (27.1)
Knowledge about clinical manifestation of thyroid disorders		
Are you of the belief that hypothyroidism frequently manifests as symptoms of coldness and weight gain?	Yes	409 (77.0)
	No	36 (6.8)
	I don’t know	86 (16.2)
Are you of believe that weight loss and feeling heated are prevalent symptoms of hyperthyroidism?	Yes	367 (69.1)
	No	40 (7.5)
	I don’t know	124 (23.4)
Is it possible for the neck tumor to indicate the presence of thyroid disorders?	Yes	379 (71.4)
	No	35 (6.6)
	I don’t know	117 (22.0)
Do you believe that fatigue may be a symptom of thyroid disorders?	Yes	430 (81.0)
	No	23 (4.3)
	I don’t know	78 (14.7)
Do you believe that diarrhea, constipation, or stomachaches may be symptoms of thyroid disorders?	Yes	210 (39.6)
	No	102 (19.2)
	I don’t know	219 (41.2)
Do you believe that alterations in the epidermis and nails may indicate the presence of thyroid disorders?	Yes	282 (53.1)
	No	57 (10.7)
	I don’t know	192 (36.2)
Could bulging eyes be indicative of thyroid disease?	Yes	259 (48.8)
	No	71 (13.4)
	I don’t know	201 (37.8)
Do you believe that bulging eyes may indicate thyroid disease?	Yes	336 (63.3)
	No	47 (8.8)
	I don’t know	148 (27.9)
Do you believe that depression may be indicative of thyroid disorders?	Yes	367 (69.1)
	No	35 (6.6)
	I don’t know	129 (24.3)
Do you believe that a delayed pregnancy may be indicative of thyroid disease?	Yes	317 (59.7)
	No	41 (7.7)
	I don’t know	173 (32.6)
Knowledge about the prevention of thyroid disorders		
Do you believe that consuming salt fortified with iodine is a preventive measure for thyroid diseases in women?	Yes	235 (44.3)
	No	57 (10.7)
	I don’t know	239 (45.0)
Can the complications of thyroid diseases be prevented by early diagnosis?	Yes	426 (80.2)
	No	20 (3.8)
	I don’t know	85 (16.0)
Do you believe that a well-balanced diet is necessary for the prevention of thyroid diseases?	Yes	392 (73.8)
	No	26 (4.9)
	I don’t know	113 (21.3)
Do you believe that refraining from consuming soy products is a preventative measure for thyroid diseases for women?	Yes	183 (34.4)
	No	53 (10.0)
	I don’t know	295 (55.6)
Do you believe that smoking cessation is an important step in the prevention of thyroid diseases?	Yes	367 (69.1)
	No	29 (5.5)
	I don’t know	135 (25.4)
Do you believe that avoiding stress is one of the ways to preventing thyroid disorders?	Yes	301 (56.7)
	No	51 (9.6)
	I don’t know	179 (33.7)
Do you believe that a well-exercised program is necessary for the prevention of thyroid diseases?	Yes	394 (74.2)
	No	33 (6.2)
	I don’t know	104 (19.6)

Table 4 presents the descriptive statistics regarding thyroid disease. The study found the mean scores of the knowledge of the risk factors, clinical manifestations, and prevention to be 3.01, 6.62, and 3.94, with mean percentages of 42.4%, 63.7%, and 57.1%, respectively. The overall mean knowledge score was 13.57, with a mean percentage of 53.8%.

**Table 4:** Descriptive statistics of the knowledge regarding risk factors, manifestation, and preventive behaviors of thyroid diseases. Descriptive statistics of the overall knowledge regarding thyroid diseases are presented in mean±SD and (%).

Variables	No. of items	Mean±SD	Mean percentage
Knowledge regarding the risk factors	8	3.01±1.32	42.4
Knowledge regarding the clinical manifestation	10	6.62±1.25	63.7
Knowledge about prevention	7	3.94±1.27	57.1
Total knowledge score	25	13.57±2.11	53.8

Table 5 shows the participants' degree of knowledge of the risk factors, symptoms, and preventive measures of thyroid diseases. The study found that the lowest knowledge score was 0, while the highest score was 25. The average score was 13.57±2.11. Out of the participants, 245 individuals (46.1%) had a low level of knowledge, with a total score below 50% (12 or less). Additionally, 224 participants (42.3%) had a moderate level of knowledge, with a total score between 50% and 75% (between 13 and 18). Lastly, 62 participants (11.6%) had a high level of knowledge, with a total score above 75% (19 or higher).

**Table 5:** Participants’ knowledge level scores of risk factors, manifestation, and preventive behaviors of thyroid diseases. Participants’ knowledge level scores are presented in frequencies (n) and proportion (%).

Knowledge level	Total scores (%)	Total scores (n)	Percentage score
Low knowledge level	Less than 50%	Score of 12 and less	46.1
Moderate knowledge level	Between 50% and 75%	Score of between 13 and 18	42.3
High knowledge level	Higher than 75%	Score of 19 and higher	11.6

Table 6 depicts the correlation between the risk factors, manifestations, and preventive behaviors of thyroid diseases and the sociodemographic characteristics of the participants. The total score for the questionnaire related to the knowledge of thyroid diseases was calculated and contrasted across various demographic categories using one-way ANOVA at varying levels of significance (p<0.05) with respect to the participants' knowledge level score. The participants' average score was 13.57±2.11. The minimal knowledge score was 0. The highest value was 25. The statistically significant difference was observed in knowledge scores based on the participant’s gender, age, education level, previous diagnosis with thyroid diseases, family history of thyroid diseases, and having performed thyroid gland investigation with p-values <0.05 (<0.001*, 0.019*, 0.027*, <0.001*, 0.023*, and 0.031*), respectively. The study found statistically higher knowledge in the following variables: being a female (p<0.001*), aged 38-48 years (p=0.019*), having university-level education (p=0.027*), previous diagnosis with thyroid diseases (p<0.001*), family history of thyroid diseases (p=0.023*), and having performed thyroid gland investigation (p=0.031*). No statistically significant differences in knowledge scores were observed based on the participants’ age, nationality, residence area, marital status, and employment status (p>0.05).

**Table 6:** The association between sociodemographic information and the knowledge score towards thyroid diseases. *Significance at p<0.05 level.

	Knowledge score (25), mean ± SD		
Variable	Category	Mean±SD	p-value
Gender	Female	15.21±1.67	<0.001*
	Male	13.13±2.57	
Age (years)	18-28	12.71±2.62	0.019*
	28-38	12.76±2.23	
	38-48	14.69±2.21	
	48-58	13.14±2.22	
	>58	12.88±1.64	
Nationality	Saudi	13.92±2.32	0.703
	Non-Saudi	12.71±2.32	
Residence area	Al-Baha city	14.95±1.81	0.061
	Other cities	12.21±2.04	
Marital status	Unmarried	15.01±2.12	0.543
	Married	15.86±1.69	
Education level	Uneducated	9.09±2.76	0.027*
	Elementary level	10.35±1.71	
	Secondary level	13.71±2.12	
	Intermediate	13.62±1.76	
	Higher education	14.28±2.14	
	University level	15.91±1.94	
Employment status	Student	13.61±1.43	0.174
	Unemployed	10.77±2.65	
	Employed	14.63±2.13	
Previous diagnosis with thyroid diseases	Yes	15.74±1.82	<0.001*
	No	12.26±2.13	
Family history of thyroid diseases	Yes	15.63±1.95	0.023*
	No	12.47±2.08	
Having performed thyroid gland investigation	Yes	15.82±1.74	0.031*
	No	12.98±1.86	

## Discussion

The study aims to assess the public's knowledge of thyroid disease manifestations and risk factors among the residents of the Al-Baha region, Saudi Arabia. The sample for this study primarily consisted of participants aged 18-28, with a predominance of females. The majority of them were married, with a university-level education, and employed. 

The results found that the prevalence of thyroid diseases among the participants was 79 (14.9%). This prevalence is considerably less than average, which is consistent with the findings by Gottwald-Hostalek and Schulte, who reported a substantially lower than one-third prevalence of thyroid diseases among the participants [11]. Comparably, the study conducted by Khan et al. reported a lower prevalence of thyroid disease, ranging between 5.1 and 5.8% among participants in Pakistan [12]. According to the findings, hypothyroidism was the most prevalent thyroid disorder reported by more than half of the participants (40, 50.6%). This finding is in agreement with that of a study conducted by Alotaibi and Almousa, which found hypothyroidism to be the most frequently reported thyroid disorder among the Riyadh population in the central region of Saudi Arabia [13]. Most participants reported that their doctor’s suggestion (91, 38.9%) inspired them to perform a thyroid function test (206, 38.8%). This is consistent with the findings by Vanderpump, who cited routine checks and doctor’s directives as reasons for participants conducting thyroid disease investigations [14].

Considering the knowledge about risk factors, more than half of the participants were aware that female gender (386, 72.7%), exposure to radiation (329, 62.0%), smoking (312, 58.8%), and family history (300, 56.5%) increase the incidence of thyroid diseases. Similarly, the study conducted by Almuzaini et al. in Saudi Arabia found family history and female gender to have an increased risk for the development of thyroid disease [15]. Comparably, the study conducted by Wiersinga found smoking as a risk factor for the development of thyroid disease [16]. Interestingly, the study revealed a need for more sufficient awareness as to amiodarone medication being a risk factor for thyroid disease, with less than one-quarter of the participants (110, 20.7%) demonstrating that knowledge. This underscores the need for public awareness campaigns and education on medications and their association with the development of thyroid diseases [16].

Regarding the clinical manifestation, most of the participants knew fatigue, feeling cold and weight gain, neck lump, and depression, with others correctly reporting hair loss to be common symptoms of thyroid diseases. The findings are consistent with those of a study conducted by Aladwani et al., which reported neck lump, fatigue, and feeling cold as the common symptoms associated with thyroid disease [17]. Moreover, the participants demonstrated good knowledge with respect to the prevention of thyroid diseases, with early diagnosis (426, 80.2%), a well-exercised program (394, 74.2%), and a well-balanced diet (392, 73.8%) reported as ways of preventing the disease and their complications. 

The findings revealed that female participants were significantly more knowledgeable about thyroid diseases than male participants, consistent with Alotaibi and Almousa, who found that female participants had higher knowledge levels towards risk factors, symptoms, and treatment of thyroid disease than their male counterparts [13]. Respondents aged 38-48 years exhibited significantly higher knowledge than other age groups. In addition, those who had a university-level education were significantly more knowledgeable regarding thyroid diseases, their risk factors, and manifestations compared to those who had a lower level of education, which is in agreement with Vanderpump, who found that participants with higher education levels exhibited good knowledge about the risk factors, symptoms, and complications related to thyroid diseases [14]. The study reported that patients diagnosed with thyroid disease previously demonstrated significantly higher knowledge than those who had not been diagnosed. Furthermore, participants who had a family history of thyroid disease were significantly more knowledgeable than those who had no family history of the disease. The findings concur with those of the study conducted by Takeuchi et al., who found that patients with close family members and relatives with a history of thyroid diseases were more knowledgeable about the disease than those without thyroid disorders [18]. Regarding the thyroid gland investigation, participants who underwent the investigation showed significantly higher knowledge about thyroid disease than those who did not. 

Utilizing a cross-sectional study design, which is capable of identifying relationships between components but not causalities, was one of the primary constraints of this investigation. Additionally, bias may have arisen due to respondents' accurate recording of their responses without a method of authenticating them, which was facilitated by the use of online surveys in the investigation. Additionally, the study's findings do not apply to the entire Saudi Arabian population due to its restriction to the Al-Baha region.

## Conclusions

Nearly half of the participants had low knowledge scores regarding the risk factors and clinical manifestations of thyroid diseases. Although a sizable proportion of participants demonstrated awareness of some clinical presentations and prevention methods, there are serious knowledge gaps regarding the risk factors and symptoms of thyroid disease among the general population in the Al-Baha region. There is a need for targeted health education programs to improve public awareness of risk factors and early detection and preventive measures of thyroid diseases to improve the quality of life among community members.

## References

[1] Kalra S, Unnikrishnan AG, Sahay R (2011). Thyroidology and public health: the challenges ahead. Indian J Endocrinol Metab.

[2] Taylor PN, Albrecht D, Scholz A, Gutierrez-Buey G, Lazarus JH, Dayan CM, Okosieme OE (2018). Global epidemiology of hyperthyroidism and hypothyroidism. Nat Rev Endocrinol.

[3] Strikić Đula I, Pleić N, Babić Leko M (2022). Epidemiology of hypothyroidism, hyperthyroidism and positive thyroid antibodies in the Croatian population. Biology (Basel).

[4] AlAwaji MI, Alhamwy RH (2023). The impact of hypothyroidism on the quality of life of adults in Riyadh, Saudi Arabia. Cureus.

[5] Alyahya A, AlNaim A, AlBahr AW, Almansour F, Elshebiny A (2021). Knowledge of thyroid disease manifestations and risk factors among residents of the Eastern Province, Saudi Arabia. Cureus.

[6] Alzahrani AS, Al Mourad M, Hafez K (2020). Diagnosis and management of hypothyroidism in Gulf Cooperation Council (GCC) countries. Adv Ther.

[7] Alqahtiani NM, Alramadhan ZT, bin Obaid MR (20201). Hypothyroidism in Saudi Arabia; prevalence, risk factors, and its relation with diabetes mellitus. Archives of Pharmacy Practice.

[8] Alshahrani RS, Mirghani H, Alharbi RT (2024). Knowledge of thyroid disease manifestation and risk factors among the general population in the Tabuk region of Saudi Arabia. Cureus.

[9] Alqahtani SM (2021). Awareness of thyroid disorders among the Saudi population. Pakistan J. Medical Health Sci.

[10] Alzahrani HS, Alshabnan RA, Mokhtar FM (2023). Assessment of Saudi society's knowledge regarding hypothyroidism and its neuropsychiatric clinical manifestations. Healthcare (Basel).

[11] Gottwald-Hostalek U, Schulte B (2022). Low awareness and under-diagnosis of hypothyroidism. Curr Med Res Opin.

[12] Khan A, Khan MA, Akhtar S (2002). Thyroid disorders, etiology and prevalence. J Med Sci.

[13] Alotaibi AMD, Almousa AIS (2018). Survey of awareness of thyroid disorders among the Riyadh population, Central Region of Saudi Arabia. Egypt J Hosp Med.

[14] Vanderpump MP (2011). The epidemiology of thyroid disease. Br Med Bull.

[15] Almuzaini A, Alshareef B, Alghamdi S (2019). Assessment of knowledge and awareness regarding thyroid disorders among Saudi people. IJMDC.

[16] Wiersinga WM (2013). Smoking and thyroid. Clin Endocrinol (Oxf).

[17] Aladwani S, Alosaimi M, Muammar M (2019). A cross-sectional survey to assess knowledge, attitude, and practices in patients with hypothyroidism in Riyadh, Saudi Arabia. Int J Pharm Res Allied Sci.

[18] Takeuchi D, Honda K, Shinohara T, Inai K, Toyohara K, Nakanishi T (2015). Incidence, clinical course, and risk factors of amiodarone-induced thyroid dysfunction in Japanese adults with congenital heart disease. Circ J.

